# Antibiofilm activity of green synthesized silver nanoparticles against biofilm associated enterococcal urinary pathogens

**DOI:** 10.1038/s41598-022-07831-y

**Published:** 2022-03-09

**Authors:** Nada S. Swidan, Yomna A. Hashem, Walid F. Elkhatib, Mahmoud A. Yassien

**Affiliations:** 1 Department of Microbiology, Faculty of Pharmacy, The British University in Egypt, Cairo, Egypt; 2 Microbiology and Immunology Department, Faculty of Pharmacy, Ain Shams University, African Union Organization St., Abbassia, Cairo, 11566 Egypt; 3 Department of Microbiology and Immunology, Faculty of Pharmacy, Galala University, New Galala city, Suez, Egypt

**Keywords:** Microbiology, Diseases, Nanoscience and technology

## Abstract

Biofilm-formed enterococcal urinary tract clinical isolates (n = 92) were used for studying the antibiofilm activity of cinnamon, ginger, and chemical AgNPs. The average particle sizes of cinnamon, ginger, and chemical AgNPs were 8.7, 41.98, and 55.7 nm, respectively. The results of Fourier transform infrared analysis revealed that phytocompounds, such as cinnamaldehyde and gingerol, were the main compounds incorporated in the synthesis of cinnamon and ginger AgNPs, respectively. The purity and crystalline nature of the AgNPs have been confirmed by energy dispersive X-ray and X-ray Diffraction analysis. The results of antimicrobial activity showed that MIC of ginger, cinnamon, and chemical AgNPs were 37.64, 725.7, and 61.08 μg/ml, respectively. On studying the antibiofilm activity of AgNPs at sub-MIC values (1/2, 1/4, and 1/8 MIC), the results revealed that it was concentration dependent. Therefore, further studies were carried out to evaluate the antibiofilm activity of AgNPs at a concentration of 18 μg/ml. The results showed that ginger and chemical AgNPs reduced the formed biofilm to 39.14% and 65.32% and the number of adherent cells on the urinary catheter surface to 42.73% and 69.84%, respectively, as compared to that of the control, while cinnamon AgNPs showed no significant activity. Accordingly, ginger AgNPs had the most potent antibacterial and antiadherent activity against biofilm-associated enterococcal isolates.

## Introduction

Biofilm is a population of bacterial cells attached to living or nonliving surfaces and surrounded by a hydrated matrix of extracellular polymeric substances (EPS)^1^. The bacterial cells enclosed in the biofilm matrix are 10–1000 times more resistant to antimicrobial agents than the planktonic ones^2^.

The National Institutes of Health reported that 80% of microbial infections in the body are caused by biofilm-associated microorganisms^3^. Biofilms can attach to several medical devices, causing persistent infections^4^. One of the medical devices associated with biofilm infections is the urinary catheter^5^. Catheter-associated urinary tract infection (CAUTI) is one of the most common nosocomial infections^6,7^.

Enterococci are Gram-positive commensals of the gastrointestinal tract and female genital organs; however, they are considered opportunistic pathogens causing several infections^8^. They are the major causative agents of CAUTI^9^. *Enterococcus faecalis* and *Enterococcus faecium* are the most common species associated with nosocomial infections, and both are known for their ability to form biofilms^7^.

Due to antibiotic resistance of biofilms, non-conventional approaches like nanoparticles were developed to combat biofilm^8^. Metal nanoparticles such as AgNPs are widely used due to their antimicrobial and antibiofilm activities. AgNPs showed antimicrobial activity by disrupting the bacterial cell membrane integrity leading to the leakage of cellular content and death^9^. In addition, AgNPs produce reactive oxygen species that interact with cellular contents as DNA, lipids, and proteins, causing malfunction in the bacteria and its death^9^.

AgNPs exert their antibiofilm activity by preventing the adhesion of bacterial cells to surfaces or by damaging the intermolecular forces^10^. It was also proven that AgNPs could inhibit quorum sensing^11^. A schematic diagram showing the antibacterial and antibiofilm activity of AgNPs is provided as Supplementary Fig. S1.

Chemical or physical methods of AgNPs synthesis are expensive and hazardous; a cost-effective and environmentally safe alternative is needed^12,13^. The synthesis of AgNPs using plant extracts has more advantages than other biological systems as being safe, low cost, easy to scale up the production, and short production time^14^. Plant extracts act as reducing agents in AgNPs synthesis and capping agents that prevent aggregation and improve the antibacterial activity of the nanoparticles^15^. For the first time in our knowledge, the green and chemically synthesized AgNPs efficacy was evaluated to combat biofilm formed by enterococcal urinary pathogens.

The current study aimed to evaluate the antibacterial and antibiofilm activity of green synthesized AgNPs using cinnamon and ginger extracts compared to chemically synthesized ones. In addition, the activity of the different AgNPs against the adherence of biofilm-associated enterococcal isolates on the surface of urinary catheters was also evaluated.

## Results

### Isolates identification

Ninety-two isolates were identified as enterococci. The results of genetic identification using PCR showed that 89% of the isolates (n = 82) were *E. faecalis,* and 11% of the isolates (n = 10) were *E. faecium* (see Supplementary Fig. S2)*.*

### Assessment of biofilm formation

The qualitative assessment of biofilm formation by tube assay showed that 94% of the isolates (n = 88) could form biofilm (see Supplementary Fig. S3). Crystal violet assay differentiated the isolates into strong (n = 23; 25%), moderate (n = 19; 21%), weak (n = 46; 50%) and non-biofilm formers (n = 4; 4%). For *E. faecalis*, 23 isolates formed strong biofilm, 18 isolates formed moderate biofilm, 39 isolates formed weak biofilm and two isolates were non-biofilm formers. On the other hand, one, seven and two *E. faecium* isolates showed moderate, weak and non-biofilm formers, respectively. All the strong biofilm producer *E. faecalis* isolates (n = 23) were selected for further studies.

### Synthesis of green silver nanoparticles

The formation of cinnamon and ginger AgNPs was indicated by changing the reaction mixture color from light yellow to dark reddish-brown and from colorless to deep yellow, respectively.

### Characterization of silver nanoparticles

#### UV–visible spectral analysis

**Figure 1:**
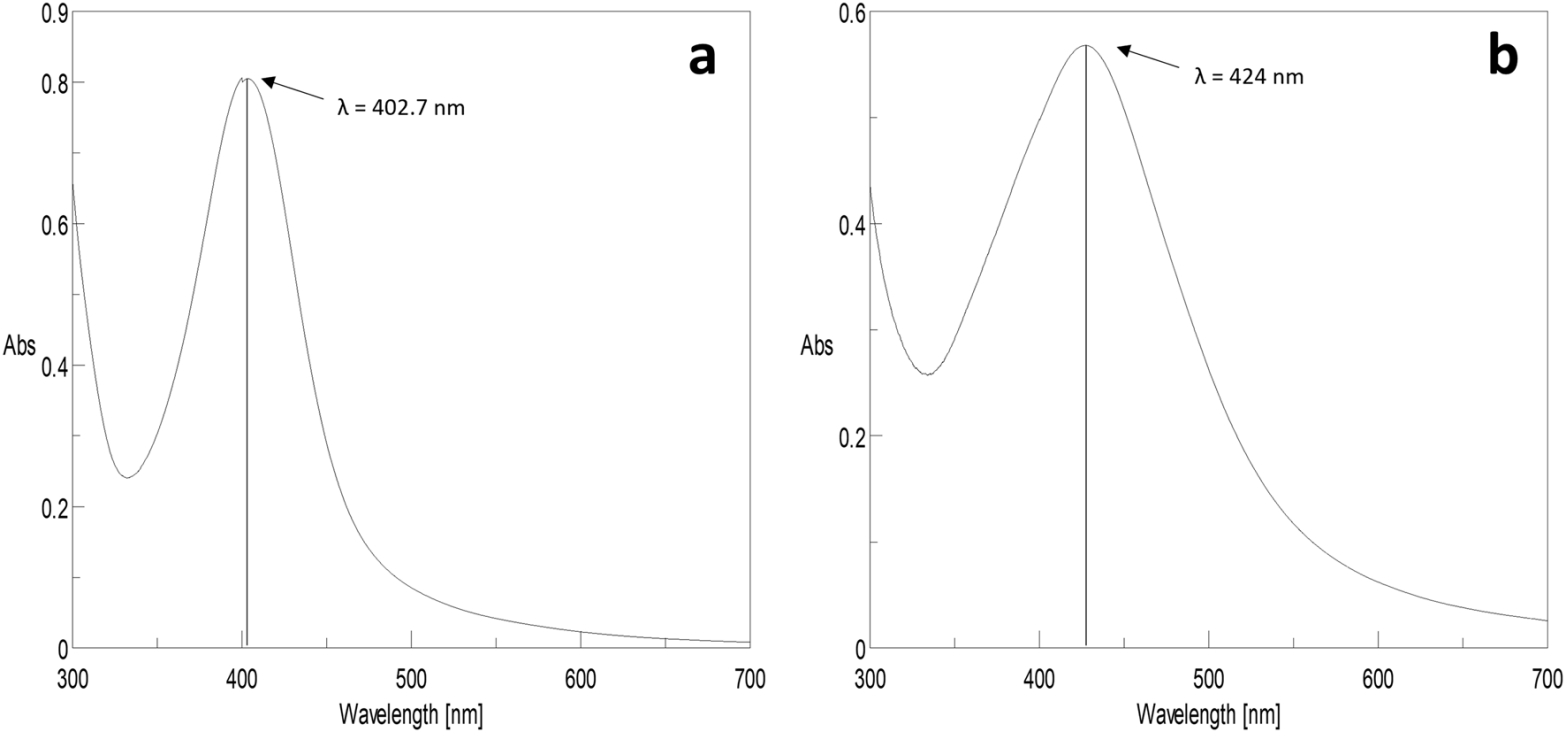
UV–Vis spectra of (**a**) cinnamon AgNPs and (**b**) ginger AgNPs.

UV–visible spectral analysis showed surface plasma resonance (SPR) band for cinnamon and ginger AgNPs at peaks around 402.7 and 424 nm, respectively (Fig. 1). The spectrum of cinnamon AgNPs was narrower than ginger AgNPs indicating that cinnamon AgNPs have a narrower particle size distribution than ginger ones.

#### Particle size and zeta potential analysis

**Figure 2:**
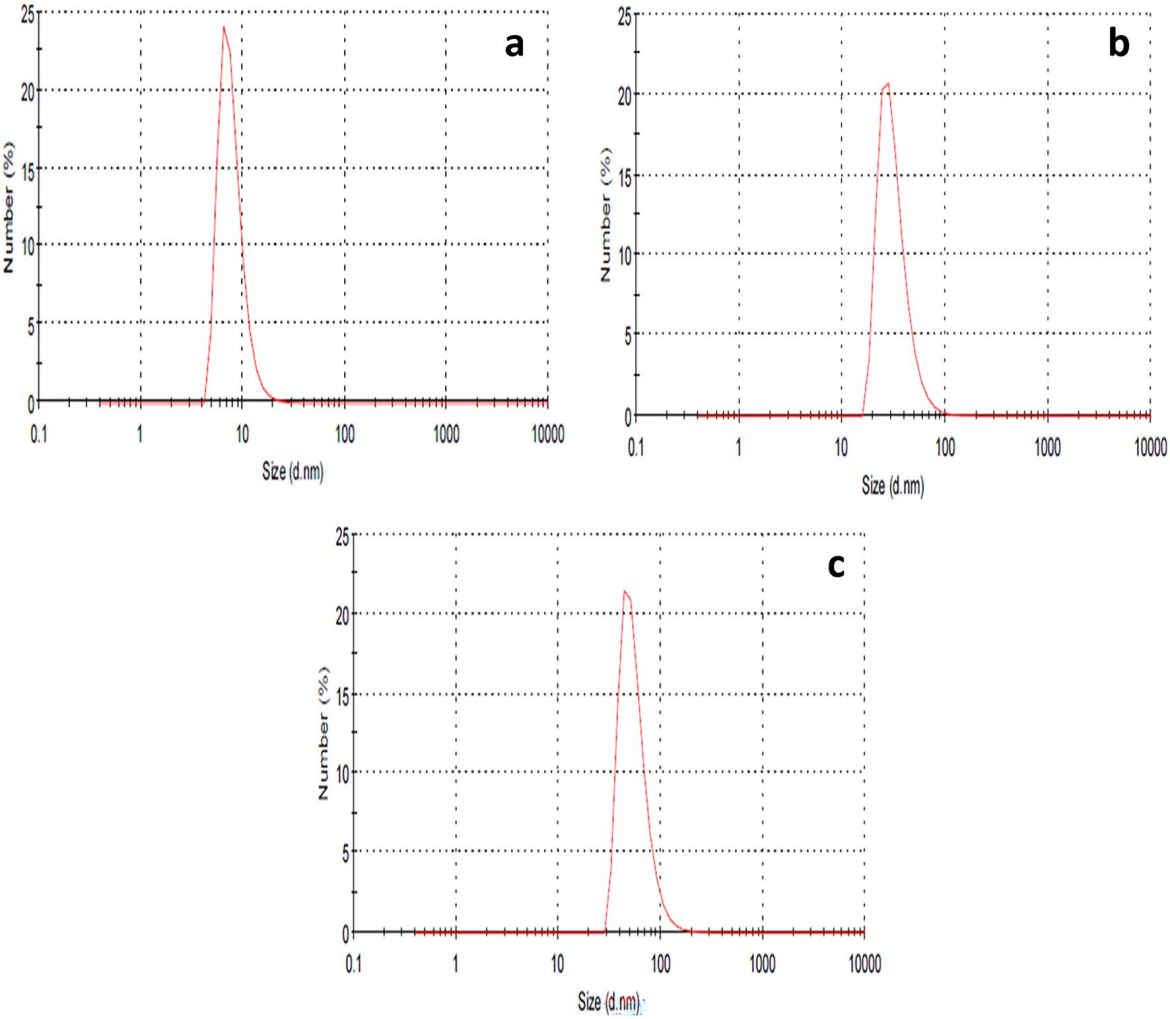
DLS images showing the particle sizes of (**a**) cinnamon AgNPs, (**b**) ginger AgNPs and (**c**) chemical AgNPs.

**Figure 3:**
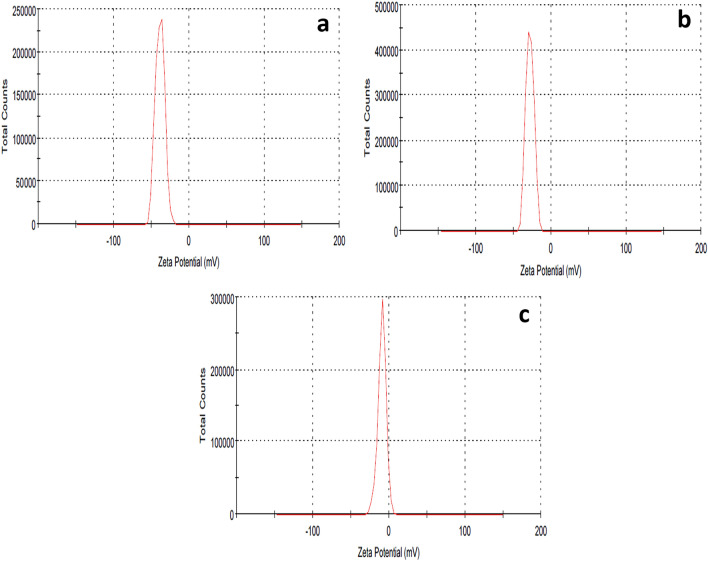
Zeta potential of (**a**) cinnamon AgNPs, (**b**) ginger AgNPs and (**c**) chemical AgNPs.

It was observed that the average particle sizes of cinnamon, ginger, and chemical AgNPs were 8.7 ± 0.7, 41.98 ± 7.2, and 55.7 ± 0.925 nm, and 0.545, 0.381, and 0.241 PdI values, respectively (Fig. 2). Histogram analysis of cinnamon, ginger, and chemical AgNPs sizes was represented as Supplementary Fig. S4. The zeta potentials of cinnamon AgNPs, ginger AgNPs, and chemically synthesized AgNPs were − 38.7, − 28.4, and − 9.35 mV, respectively (Fig. 3).

#### Fourier Transform Infrared (FT-IR) analysis

**Figure 4:**
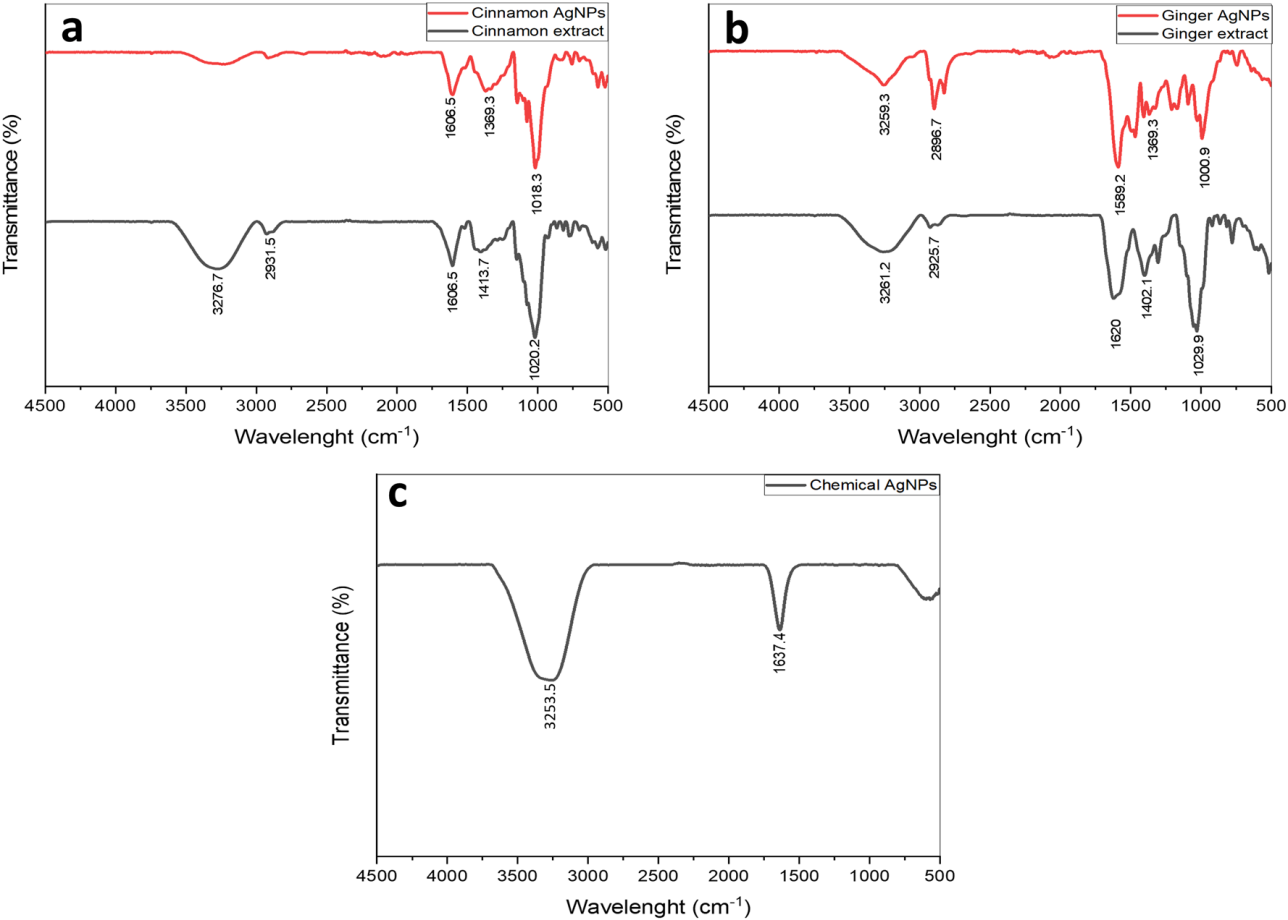
FTIR spectra of (**a**) cinnamon extract (black) and cinnamon AgNPs (red), (**b**) ginger extract (black) and ginger AgNPs (red), and (**c**) chemical AgNPs.

Functional groups of compounds used in the synthesis of AgNPs were identified by Fourier transform infrared (FT-IR) analysis (Fig. 4). The cinnamon AgNPs FT-IR spectrum showed peaks at 1606.5, 1369.3, and 1018.3 cm^−1^, corresponding to C=O, C=C, and C–O groups, respectively. These peaks were similar to those in the cinnamon extract FT-IR spectrum. In ginger AgNPs FT-IR spectrums, a peak at 3259.3 cm^−1^ was assigned to the O–H stretching and at 2896.7 cm^−1^ to the C–H group stretching of aromatic compounds. In addition, peaks at 1589.2, 1369.3, and 1000.9 cm^−1^ were attributed to the stretching of C=O, C=C, and C–O groups. The peaks that appeared in the ginger AgNPs spectrum were equivalent to those in the ginger extract FT-IR spectrum. The chemically synthesized AgNPs FT-IR spectrum showed peaks at 3253.5 and 1637.4 cm^−1^ related to the O–H stretching/N–H stretching of an amine group and C=O groups, respectively.

#### Energy dispersive X-ray analysis

**Figure 5:**
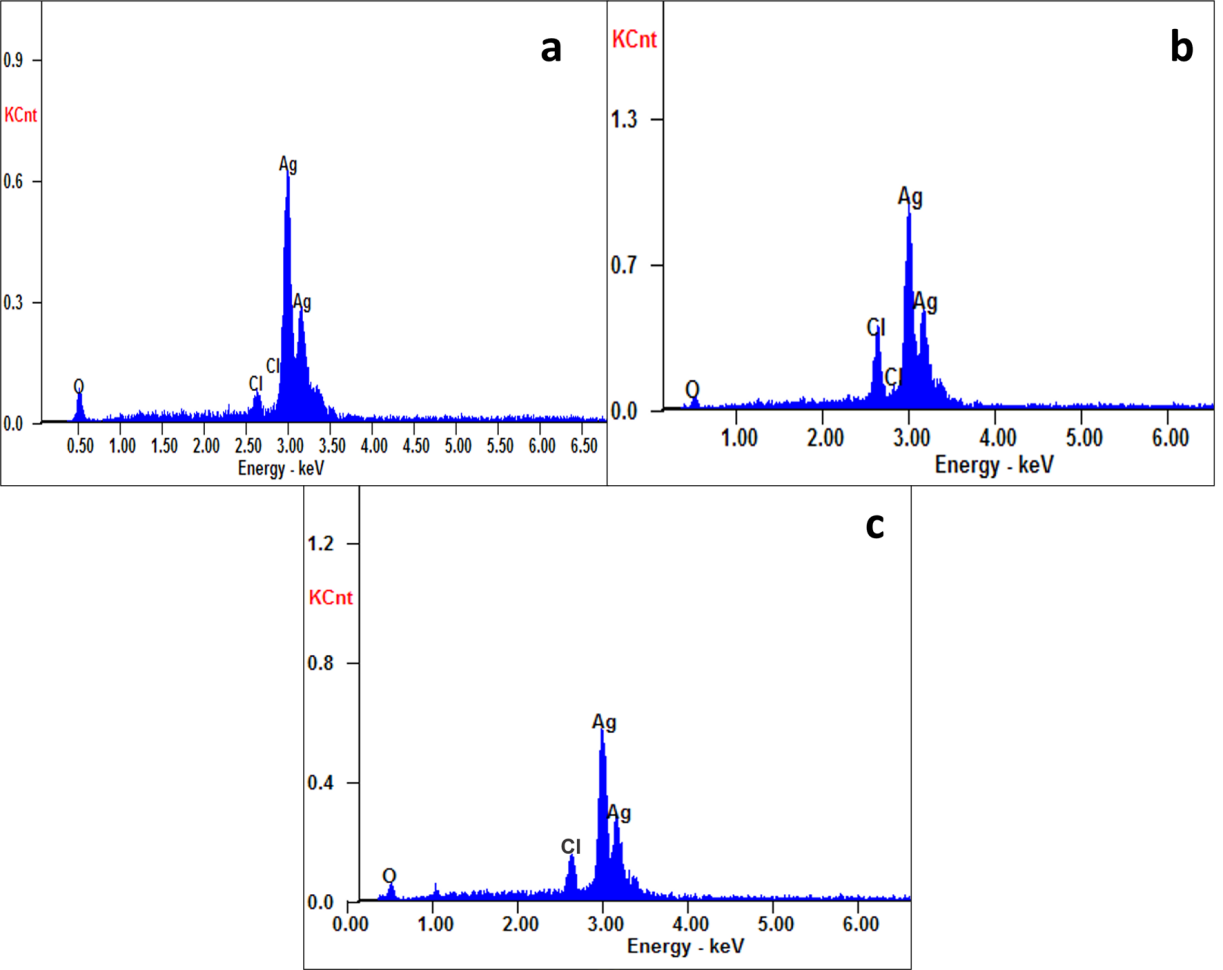
EDX spectra of (**a**) cinnamon AgNPs, (**b**) ginger AgNPs, and (**c**) chemical AgNPs.

The energy dispersive X-ray (EDX) analysis was performed to test the purity and the elemental composition of the synthesized AgNPs. Strong signals at 3 and 3.2 keV were noticed in the EDX pattern of the AgNPs corresponding to elemental silver, indicating the complete reduction of silver ions (Fig. 5). Ginger AgNPs showed the highest concentration of elemental silver compared to cinnamon AgNPs and chemically synthesized AgNPs (Table 1). Weak signals for oxygen and chlorine were revealed in the EDX pattern with a low percentage as they acted as capping agents on the nanoparticles.

**Table 1:** EDX elemental analysis of cinnamon, ginger, and chemical AgNPs.

	Silver, %	Chlorine, %	Oxygen, %
Cinnamon AgNPs	74.08	1.15	24.77
Ginger AgNPs	79.68	8.3	12.02
Chemical AgNPs	70.97	5.2	23.07

#### X-ray diffraction (XRD) analysis

**Figure 6:**
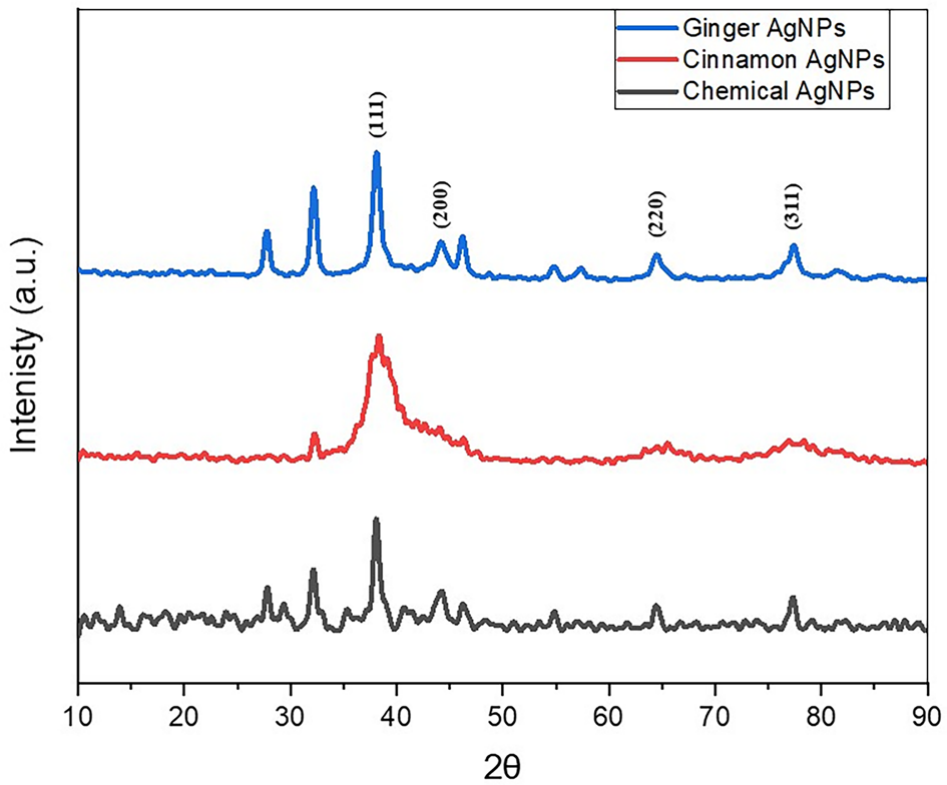
XRD pattern of ginger AgNPs (blue), cinnamon AgNPs (red) and chemical AgNPs (black).

The Crystalline characterization of the green and chemically synthesized AgNPs was done using XRD (Fig. 6). The Diffraction pattern of cinnamon AgNPs showed peaks at 38.27°, 65.38°, and 77.53°, corresponding to (111), (220), and (311) plane lattice. In addition, ginger and chemical AgNPs showed diffraction angles at 38.1°, 44°, 64.3°, and 77.2° with lattice planes of (111), (200), (220), and (311). According to the ICDD PDF-2 (No.071-4613) database, these lattice planes were indexed to the face-centered cubic crystal (FCC) nature of metallic silver. The average crystallite size of the AgNPs was calculated by applying the Scherrer equation^16^. Cinnamon, ginger, and chemical AgNPs' average crystallite sizes were 5.7, 10.3, and 14.6 nm, respectively.

#### Transmission electron microscope (TEM)

**Figure 7:**
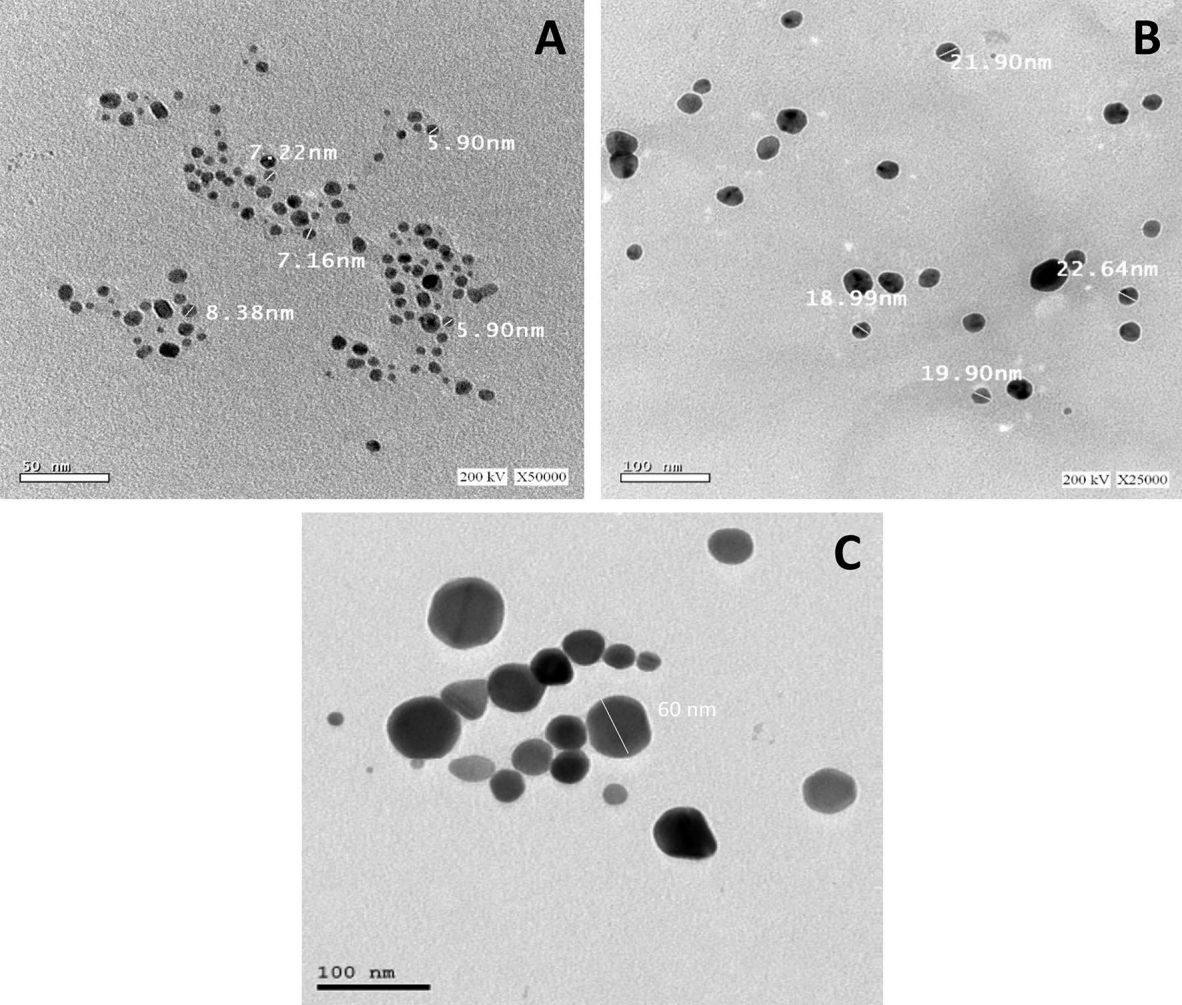
TEM analyses of synthesized AgNPs (**a**) TEM micrograph of cinnamon AgNPs, (**b**) TEM micrograph of ginger AgNPs and (**c**) TEM micrograph of chemical AgNPs.

TEM results showed that cinnamon AgNPs, ginger AgNPs, and chemically synthesized AgNPs were spherical, monocrystalline, and well-distributed. The average particle sizes of cinnamon and ginger AgNPs were 6.3 and 20.2 nm, respectively (Fig. 7). Chemically synthesized AgNPs were the largest, with an average particle size of 60 nm (Fig. 7).

#### Antibacterial effect of silver nanoparticles

The MICs ranges of cinnamon, ginger, and chemical AgNPs using broth microdilution were 156–1250, 15.63–62.5, and 31.25–62.5 μg/ml with mean values 725.7, 37.64, and 61.08 μg/ml, respectively. Student’s *t*-test was performed between MICs values of every two AgNPs. It was observed that there was a significant difference between the MICs of the three types of AgNPs with *p-*values less than 0.0001. The MBC ranges cinnamon, ginger, and chemical AgNPs were 156–1250, 15.63–125, and 62.5–125 μg/ml with mean values 725.7, 87.64, and 100.54 μg/ml, respectively.

Further studies were carried out to evaluate the antibacterial activity of cinnamon and ginger extracts and AgNO_3_. The results showed that MICs of both cinnamon and ginger extracts were > 4 mg/ml, while that of AgNO_3_ was 62.5 µg/ml.

### Antibiofilm assay

#### Microtiter plate assay

**Figure 8:**
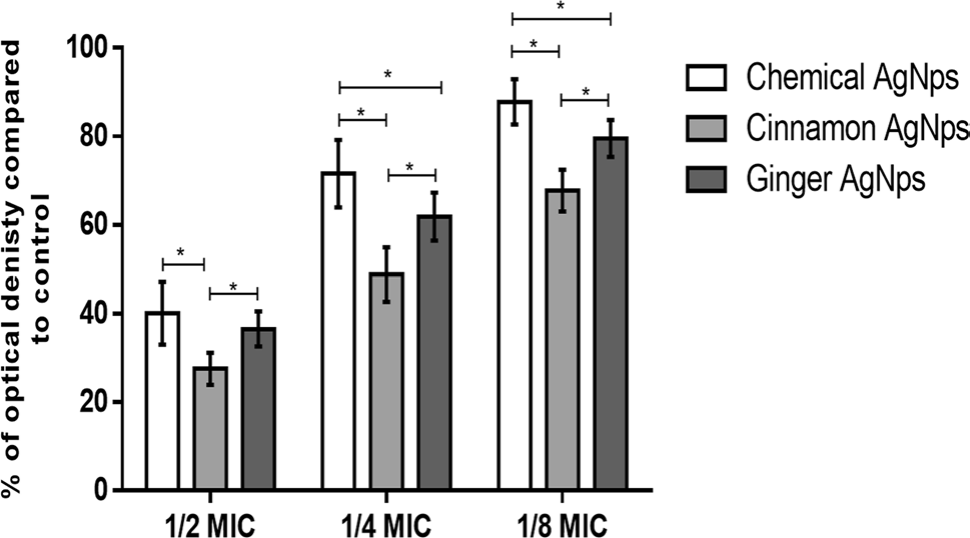
A comparative analysis of % of optical density of formed biofilm against different concentrations of cinnamon AgNPs, ginger AgNPs, and chemical AgNPs using two-way ANOVA test (Data are presented as mean ± SD, n = 23, *p*-value (*) < 0.0001).

The obtained results showed that the average of the percentages of biofilm formed in the presence of 1/2 MIC of cinnamon, ginger, and chemical AgNPs was 27.85%, 36.7%, and 40%, respectively, compared to that of the control. The 1/4 MIC of cinnamon, ginger, and chemical AgNPs reduced the biofilm formation to 49.23%, 62.6%, and 71.59%, respectively. The lowest reduction of the biofilm formation was noticed in the presence of 1/8 MIC of cinnamon, ginger, and chemical AgNPs, in which percentages of biofilm formed compared to the control were 68.11%, 79.53%, and 87.48%, respectively (Fig. 8). A two-way ANOVA test was applied between the different concentrations of different AgNPs against the percentage of biofilm formed. At 1/2 MIC levels, the percentage of biofilm formed in the presence of cinnamon AgNPs was significantly less than that formed in chemical and ginger AgNPs (*p* < 0.0001). There was no significant difference between the percentage of biofilm formed in the presence of ginger AgNPs and chemical AgNPs (*p* = 0.0832) at 1/2 MIC levels. At 1/4 and 1/8 MIC, ginger AgNPs showed a significantly lower percentage of biofilm formation than chemical AgNPs (*p* < 0.0001).

According to the obtained results, the antibiofilm effect of the tested AgNPs was concentration dependent. Therefore, further studies were carried out to evaluate their activity at constant concentration (18 μg/ml). The obtained results showed that the use of ginger and chemical AgNPs at concentration 18 μg/ml reduced the adherent biofilm to 39.14% and 65.32%, as compared to the control, respectively. While in the case of cinnamon AgNPs, no significant reduction in the formed biofilm was observed.

Further studies were carried out to evaluate antibiofilm of cinnamon and ginger extracts and AgNO_3_ at sub-MIC values. According to the obtained results, no significant changes in the adherent biofilms was determined in the presence of cinnamon and ginger extracts and AgNO_3_ at concentration 125, 62.5, and 15.625 µg/ml or less, respectively. Also, on studying the antibiofilm activity of both extracts and AgNO_3_ at concentration 18 µg/ml, no change in the biofilm formation was observed as compared to the control.

### Antibiofilm effect of AgNPs on the catheter

**Figure 9:**
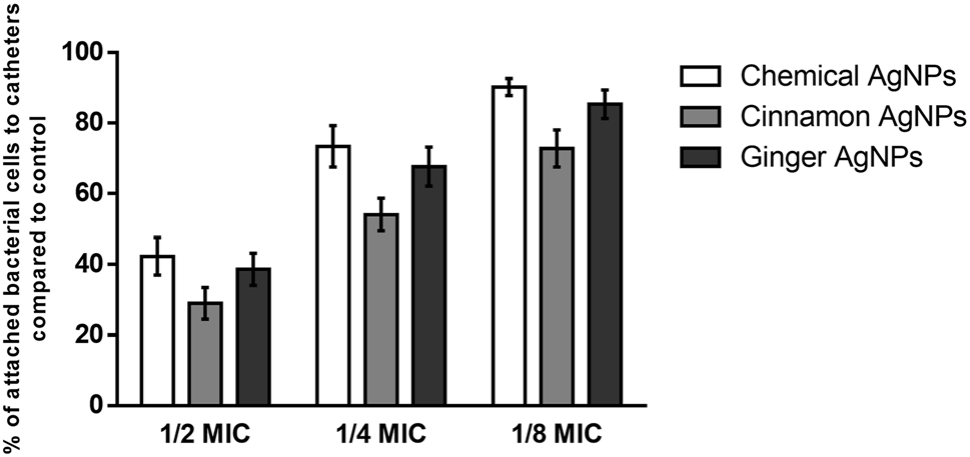
A comparative analysis of % of attached bacterial cells on catheter surfaces compared to control for cinnamon AgNPs, ginger AgNPs, and chemical AgNPs (Data are presented as mean ± SD, n = 23).

The results of the effect of cinnamon, ginger, and chemical AgNPs at 1/2, 1/4, and 1/8 MIC of AgNPs on the adherence of enterococcal isolates were presented in Fig. 9. In the presence of 1/2 MIC, the average count of the adhered enterococcal cells was reduced to 29%, 38.6%, and 42% as compared to the control, respectively. The counts of adhered cells at 1/4 MIC of cinnamon, ginger, and chemical AgNPs were reduced to 54%, 67.67%, and 73.4% as compared to the control, respectively. While in the presence of 1/8 of MIC of the AgNPs, the count adherent cells reduced to 72.77%, 85.3%, and 90.2% as compared to the control, respectively.

Further studies were carried out to evaluate the anti-adherent activity of cinnamon, ginger, and chemical AgNPs using a constant concentration (18 μg/ml). The obtained results showed that ginger and chemical AgNPs caused a reduction in the adherent enterococcal cells to 42.73% and 69.84%, respectively. On the other hand, cinnamon AgNPs showed no reduction in the number of adherent cells.

### Scanning electron microscope of catheters

**Figure 10:**
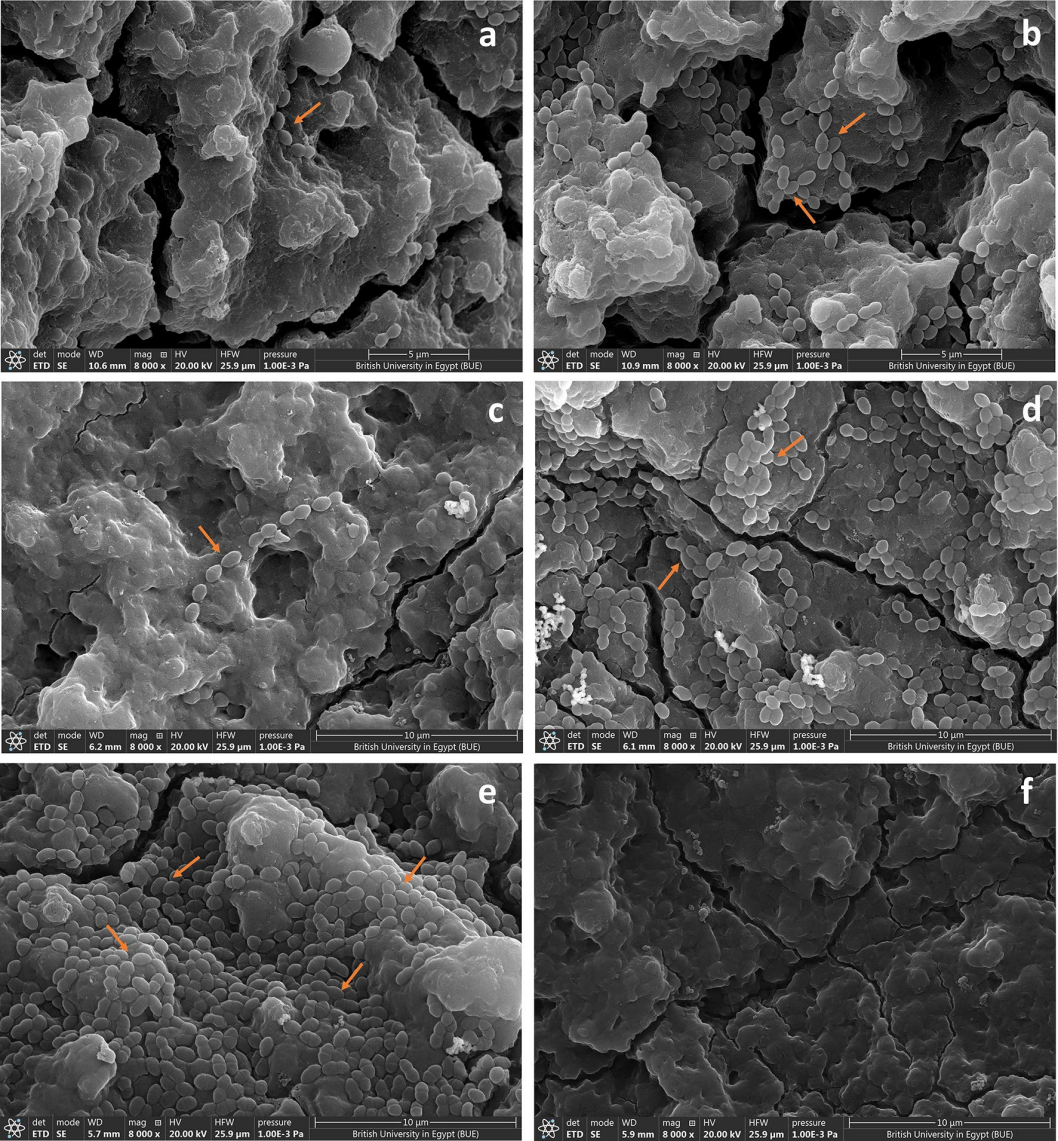
SEM images showing a very few scattered enterococcal cells adhered on the catheter surface treated with (**a**) ½ MIC ginger AgNPs and (**c**) ½ MIC chemical AgNP, and a reduction in the number of the adherent enterococcal cells on catheters treated with (**b**) ¼ MIC ginger AgNPs and (**d**) ¼ MIC chemical AgNPs compared to (**e**) positive control catheter that showed a heavy accumulation of enterococcal cells on the surface of the catheters. (**f**) Catheter free from bacterial cells (negative control).

The SEM image of the positive control catheter segment showed bacterial biofilm heavy accumulation of the adherent cells. In case of the catheter segments treated with ½ MIC of ginger or chemical AgNPs, few scattered adherent cells were observed. The number of the adherent cells was increased in case of the catheter segments that were treated with ¼ MIC of ginger or chemical AgNPs, but still much less than that of the positive control. The surface of the negative catheter segments showed no adherent cell (Fig. 10a–f).

## Discussion

Biofilms are associated with severe health problems, especially when they bind to medical devices such as urinary catheters, leading to persistent infections^17^. One of the main pathogenic agents that cause CAUTIs is enterococci^18^. Resistance associated with biofilm necessitates finding effective alternatives^19^. Green synthesized AgNPs are new, safe, and non-toxic approach to eradicate biofilm^20^.

In the present study, a total of 92 enterococcal clinical isolates were identified, and the majority of them (n = 82) were *E. faecalis.* This result was in agreement with that investigated by Hashem et al*.* who found that most enterococcal isolates from patients with UTI were identified as *E. faecalis*^21^. The majority of tested isolates (94%; n = 88) were capable of forming biofilm, similar to that reported by previous studies^22,23^.

Green AgNPs were synthesized, in the present study, by cinnamon bark and ginger root extracts as they act as reducing and stabilizing agents^15^. The formation of AgNPs was indicated by changing the color of silver nitrate solution from light yellow to dark reddish-brown for cinnamon AgNPs and from colorless to deep yellow for ginger AgNPs. These findings were compatible with previous studies that synthesized cinnamon and ginger AgNPs^24,25^. UV–visible spectroscopy was used as an effective way to confirm the synthesis of AgNPs^16^. Cinnamon and ginger AgNPs showed SPR peaks around 402.7 and 424 nm, respectively. The obtained results agreed with previous studies done by Sathishkuma et al*.* and Yang et al*.* that reported that cinnamon and ginger AgNPs showed bands with peaks at 426 nm and 423 nm, respectively^24,26^. Comparing our findings with the previous results, it was revealed that both cinnamon and ginger AgNPs were synthesized.

Cinnamon AgNPs showed smaller particle size than ginger AgNPs as the SPR peak of cinnamon AgNPs showed a shorter wavelength than ginger AgNPs. In addition, the cinnamon AgNPs had a narrower particle size distribution than the ginger ones, and this was confirmed by the calculated standard deviation (SD) resulting from particle size measurement by DLS. The DLS results confirmed the interpretation of UV-spectra of both cinnamon and ginger AgNPs. It was noticed that the green synthesized AgNPs were smaller particle size than the chemically synthesized ones. Our results agreed with Anjum et al., who synthesized cinnamon AgNPs with particle size range 5–25 nm^27^. In addition, ginger AgNPs synthesized in this study showed smaller particle size than the ginger AgNPs synthesized by Reda et al.^28^. The zeta potential values determined the stability of AgNPs; as the zeta potential magnitude increases, the repulsion force between the nanoparticles increases, thus nanoparticles' stability increases^29^. Comparing the zeta potential values, cinnamon AgNPs were more stable than ginger AgNPs, and the least stability was shown with chemically synthesized AgNPs. TEM results confirmed the results shown by DLS.

Identification of the functional groups of compounds that were incorporated in the AgNPs’ synthesis was carried out by FT-IR. It was revealed that C=O, C=C, and C–O groups from phytocompounds found in cinnamon and ginger extracts were used in the synthesis of cinnamon and ginger AgNPs. The expected major phytocompounds that played as capping and reducing agents in the green synthesis of cinnamon and ginger AgNPs were cinnamaldehyde and gingerol, respectively^27,30^. These phytocompounds may play a role in stabilizing the particle size of the prepared silver metals within the nanoparticle ranges. Similar FT-IR results were reported in previous studies of green synthesized AgNPs^16,31,32^. The chemical AgNPs FT-IR spectrum peaks were corresponding to the O–H stretching/N–H stretching of an amine group and C=O groups. This result proved that chemical AgNPs were synthesized using polyvinylpyrrolidone (PVP). The FT-IR results of chemical AgNPs agreed with previous studies that showed peaks at similar wavelengths^33,34^.

The purity of the green and chemically synthesized AgNPs were confirmed by EDX analysis. The strong and sharp signals, observed at 3 and 3.2 keV, were referred to elemental silver. It was reported previously that a high signal at 3 keV confirmed the presence of AgNPs^35^. In addition, oxygen atom signal was observed in EDX pattern. These oxygen atoms were from the plant extracts and acted as a capping agent to AgNPs^35^. The weight percentages of silver in cinnamon, ginger, and chemical AgNPs revealed from the EDX pattern were 74.08, 79.68, and 70.97%, respectively, in agreement with that reported by Otunola et al*.* (79.27%) for ginger AgNPs^31^.

The crystalline nature of AgNPs exhibited the FCC crystal nature of metallic silver as (111), (200), (220), and (311) plane lattice observed in the XRD pattern of ginger and chemical AgNPs and (111), (200), and (311) plane lattice in XRD pattern of cinnamon AgNPs. Similar XRD pattern was observed in previous studies of green synthesized AgNPs^32,35^.

The green and chemically synthesized AgNPs were tested for their antimicrobial activity against the selected strong biofilm producer enterococcal urinary isolates (n = 23). The broth microdilution method showed a MIC range of 31.25–62.5 µg/ml for chemical AgNPs. In a previous study, 1000 µg/ml of chemically synthesized AgNPs inhibited the growth of *E. faecium* for the first 6 h, and after that, slight growth occurred^36^. Krishnan and his coworkers reported that chemically synthesized AgNPs showed a MIC of 5000 µg/ml against *E. faecalis*^37^. Regarding the green synthesized AgNPs, the MIC ranges were 156–1250 and 15.6–62.5 µg/ml for cinnamon, and ginger AgNPs, respectively. The MIC of ginger AgNPs against enterococci isolates was significantly lower than that of the other nanoparticles (*p* < 0.0001). Therefore, green synthesized AgNPs, especially ginger AgNPs, were more potent antibacterial agents than chemical and cinnamon AgNPs. It was previously reported that AgNPs synthesized from *Rhododendron ponticum* and *Mimusops elengi* showed MIC values against *E. faecalis* at 250 and 312.5 µg/ml, respectively^38,39^. Rashnaei and his coworkers reported that *E. faecalis* showed the highest resistance to the anise AgNPs at concentration 100 µg/ml^40^. The preference of green synthesized AgNPs as antibacterial agents over chemically synthesized ones was in agreement with the study done by Mousavi-Khattat et al., who reported that green synthesized AgNPs from *D. stramonium* seeds were more effective antibacterial agents against *Escherichia coli* (ATCC 25922) and *Staphylococcus aureus* (ATCC 33591) than chemically synthesized AgNPs^41^. In addition, Shaik et al. reported that AgNPs synthesized using miswak extract showed higher antibacterial effect than chemically synthesized ones against Gram-positive and negative bacteria^42^.

The antibacterial activity of the used extracts (cinnamon and ginger) and AgNO_3_ was evaluated to clarify their effect in the developed antibacterial activity of the prepared AgNPs. According to the obtained results, both extracts had MIC values (> 4 mg/ml) higher than that of the corresponding AgNPs indicating that the antibacterial activity of the prepared green AgNPs did not depend on cinnamon or ginger extract in their antibacterial activity. Also, it should be clarified that during the process of green AgNPs preparation, a complete elimination of the residual AgNO_3_ and both plant extracts from the AgNPs product was carried out by washing. In addition, the purity of the green and chemically synthesized AgNPs were confirmed by EDX analysis.

The sub-MICs of the selected AgNPs were tested against the strong biofilm-forming enterococcal isolates. It was revealed that the three forms of AgNPs had antibiofilm activity against the isolates with variation in their strength. Upon using the same sub-MIC level, the results showed that cinnamon AgNPs significantly (p < 0.0001) inhibited biofilm more than the other AgNPs, but with higher concentration. There was no statistical difference (*p* = 0.0832) between the decrease of biofilm formed in the presence of ginger AgNPs at 1/2 MIC level and that formed in the presence of chemical AgNPs at the same concentration level; however, the 1/2 MIC numerical value of ginger AgNPs was significantly (*p* < 0.0001) lower than that of chemical AgNPs. The percentage of biofilm formed in the presence of 1/4 and 1/8 MIC levels of ginger AgNPs was significantly (*p* < 0.0001) lower than that formed at the same concentration level of chemical AgNPs.

According to the obtained results, the antibiofilm activity of the tested AgNPs was concentration dependent. This observation was confirmed by the SEM images of urinary catheters treated with ginger and chemical AgNPs and compared with that of the control. In addition, it was in agreement with that reported by Seo et al.^43^. Therefore, further studies were carried to investigate the antibiofilm activity of the tested AgNPs using the same concentration (18 µg/ml). The obtained results revealed that ginger AgNPs had more antibiofilm activity than chemical AgNPs. While cinnamon AgNPs showed no antibiofilm activity. This observation was confirmed by the results of the efficacy of the AgNPs in preventing the adherence of enterococcal cells on the urinary catheters.

The effect of the used extracts (cinnamon and ginger) in the antibiofilm activity of the prepared green synthesized AgNPs was determined by testing their antibiofilm activity against the used enterococcal isolates. The obtained results revealed that cinnamon and ginger extracts had no antibiofilm activity at concentrations 125 and 62.5 µg/ml or less, respectively as compared to the control. In addition, complete elimination of the remaining plant extracts and AgNO_3_ after preparing green synthesized AgNPs was carried out by washing. These results confirmed that the antibiofilm activity of the tested AgNPs were mainly due to the newly formulated AgNPs.

Accordingly, ginger AgNPs were more potent antibiofilm agents than cinnamon and chemical AgNPs. The priority of green synthesized AgNPs over chemical AgNPs on inhibiting the biofilm formed by *Staphylococcus aureus* was reported previously by Barabadi et al.^44^. Furthermore, AgNPs synthesized from *Artemisia scoporia* extract exhibit more antibiofilm activity against multidrug-resistant *S. aureus* than chemical AgNPs^45^.

The hazard of AgNPs coated on urinary catheters was tested by Chutrakulwong et al., who *reported* that the amount of silver liberated from urinary catheter coated with AgNPs over 10 days was about 0.040 µg/ml, which is much less than that leads to toxicity ( 44 µg/ml)^46^. In addition, the coated catheter with AgNPs was introduced subcutaneously in mice, and after 10 days, Roe et al*.* didn’t observe inflammation at the site of implantation nor any toxicity signs^47^.

## Conclusion and future studies

In this study, the green synthesized AgNPs, especially those synthesized from ginger extract, aren’t only less hazardous, cost-effective, simple, fast, and easy to scale up but also improve the size, stability, antimicrobial and antibiofilm activity of the nanoparticles against enterococcal urinary pathogens. Various techniques, including UV visible, XRD, FT-IR spectroscopy, EDX, and TEM, confirmed the synthesis of pure and stabilized AgNPs from plant extracts. The ginger AgNPs exerted more antibacterial activity than cinnamon and chemical AgNPs. In addition, ginger AgNPs were more efficient in preventing the adherence of strong biofilm-forming enterococcal cells on the surface of the urinary catheter than cinnamon and chemical AgNPs. For determination of the clinical relevance of these experimental data, a well-controlled in-vivo and clinical trial are needed.

## Methods

### Isolates collection and identification

Ninety-two isolates were collected from Al-Borg Medical Laboratories and El-Shatby Hospital in Egypt from patients with urinary tract infections (UTI) from 2018 to 2019. Identification of isolates to genus level was done by surface streaking on Bile Esculin agar (Biokar diagnostics, France) and chromogenic UTI agar (Conda, Spain) followed by Gram stain for pure colonies. Biochemical tests were done to confirm enterococcal identification as NaCl tolerance test and catalase test.

### Identification to species level

Identification of enterococcal isolates to species level was performed by polymerase chain reaction (PCR) using *ddl* primers. Primers used for *E. faecalis* identification were 5′ATCAAGTACAGTTAGTCTTTA-3′ and 5′-AACGATTCAAAGCTAACT-3′, and for *E. faecium* were 5′-CCAAGGCTTCTTAGAGA-3′ and 5′-CATCGTGTAAGCTAACTTC-3′^48^. PCR was done using 25 µl Dream Taq Green PCR Master Mix (Thermo Scientific, USA), 0.1 µl from each primer, 1 µg of the extracted DNA and to 50 µl with RNase free water PCR products were analyzed by gel electrophoresis and visualized under UV light. The PCR product size of 942 and 535 bp indicated the presence of *E. faecalis* and *E. faecium*, respectively.

### Assessment of biofilm formation

#### Tube method

The tube method is a qualitative method for biofilm detection. A loopful of an overnight culture of the tested organism was inoculated in 5 ml of sterile Trypticase Soy Broth (Oxoid, UK) supplemented with 1% w/v glucose (TSBG). The tubes were incubated at 37 °C for 24 h. After incubation, tubes were decanted, washed with phosphate-buffered saline (PBS; pH 7.3) to remove planktonic free bacterial cells, and left to dry. Tubes were then stained with crystal violet (0.1% w/v) (Oxford, UK). The excess stain was washed with distilled water, and the tubes were left to dry. The formed violet layer on the inner walls and bottom of the test tube indicated biofilm formation^49^.

#### Crystal violet assay

Crystal violet assay was done according to Christensen et al. with modification. A loopful of an overnight culture of the tested organism was inoculated in a test tube containing 5 ml of sterile TSBG and incubated at 37 °C overnight. Culture density was adjusted to 0.5 McFarland using spectrophotometer V-630 (Unicam, UK), then diluted 1:100 using TSBG to achieve a final concentration of 10^6^ CFU/ml. Sterile flat bottomed 96 well plates were inoculated with 200 μl from each diluted culture, and this was done in triplicate, then incubated at 37 °C for 24 h. Wells containing bacteria free media were used as controls.

After incubation, contents of each well were discarded, plates were washed with 200 μl PBS three times and left to dry. Adherent bacterial cells were fixed by methanol and stained by adding 150 μl of crystal violet (0.1% w/v) (Oxford, UK). Excess stain was removed, and plates were washed and left to dry. The adhered bacterial cells were resolubilized by 33% glacial acetic acid (Fisher Chemical, US). The plates' optical density (OD) was measured by plate reader ELx800 (Biotek, USA) at wavelength 630 nm. The averages of three optical densities were taken, and the standard deviation was calculated^49,50^.

The strength of biofilm was classified according to OD reading as follows: O.D. ≤ O.D.c (O.D. of the negative control) = non-adherent, O.D.c < O.D. ≤ (2 × O.D.c) = weakly adherent, (2 × O.D.c) < O.D. ≤ (4 × O.D.c) = moderately adherent and (4 × O.D.c) < O.D. = strongly adherent^51^.

### Synthesis of silver nanoparticles

*Cinnamon cassia* barks and *Zingiber officinale* root were purchased from Egypt's local market. Cinnamon AgNPs were synthesized as follows: the cinnamon extract was prepared by placing 2.5 gm of the *Cinnamon cassia* barks powder in a flask containing 100 ml of distilled water and stirred for 15 min at 70 °C. Then, the extract was filtered, and the collected filtered was centrifuged at 15,294×*g* for 15 min. A 100 ml solution (1 mM) of AgNO_3_ (Fisher Chemical, USA) was heated to 70 °C with vigorous stirring in dark condition. An aliquot of prepared cinnamon extract (10 ml) was added to the AgNO_3_ solution dropwise (final pH was 11). Changing the color of the mixture from light yellow to dark reddish-brown was an indication of nanoparticles formation^27^. Collection and purification of the formed nanoparticles were carried out according to Moodley et al*.* with modification by centrifugation of the colloidal solution at 15,294×*g* for 3 h at 4 °C using 2-16KL centrifuge (Centurion, UK)^52^. The collected precipitate was washed thrice with distilled water^52^.

Ginger AgNPs were prepared by the same procedure as described in cinnamon AgNPs with slight modification. The extract was prepared by using 20 gm *Zingiber officinale* root in 100 ml of distilled water. Changing the color of the mixture solution from colorless to deep yellow was an indication of nanoparticles formation (final pH was 6). Separation of the formed nanoparticles was carried out by centrifugation for 40 min^28^. The steps and mechanism of green AgNPs synthesis were illustrated in a schematic diagram as Supplementary Fig. S5.

Chemically synthesized AgNPs were purchased from Nanotech Company (6 October city, Egypt) as a solution of 1 mg/ml concentration. The purchased AgNPs were synthesized by using PVP as a capping agent.

### Characterization of silver nanoparticles

The formation of green synthesized AgNPs was confirmed by a UV visible spectrophotometer V-630 (Unicam, UK), and the absorption spectrum was adjusted from 300 to 700 nm. The particle size and zeta potential of the different AgNPs were measured using Zetasizer Nano ZS (Malvern Instruments, UK). The Bruker Vertex 70 FT-IR (Germany) was used with a resolution of 4 cm^−1^ at 500–4500 cm^−1^ in the attenuated total reflection (ATR) mode to identify various organic compounds were responsible for silver ion reduction and capping of green and chemically synthesized AgNPs. The elemental composition and the purity of the AgNPs were determined by EDX analysis using FEI Inspect S scanning electron microscope (SEM–EDX) (Netherland). In order to identify the crystalline nature of the AgNPs, XRD was performed by Malvern Panalytical Empyrean 3 (Netherland). The XRD operation was run at 40 kV and 30 mA with Cu Kα radiation of 1.54 A° wavelength, in the 2θ range 10°–90° angle. In addition, the Scherrer equation was used to calculate the average crystallite sizes of the AgNPs^16^. A high-resolution transmission electron microscope (JEOL-JEM2100, Japan) (200 kV) was used to confirm the particle size and study the shape of the AgNPs.

### Antibacterial effect of AgNPs

In 96 well microtiter plate, serial dilutions of cinnamon AgNPs (5000–39 μg/ml), ginger AgNPs (1000–7.8 μg/ml), and chemical AgNPs (1000–7.8 μg/ml) were done separately using TSBG, and 100 μl of the bacterial suspension (10^6^ CFU/ml, prepared as described before) was added to each well. Positive control, including bacteria and media, and negative control, including media only, were included. The plates were incubated at 37 °C for 24 h. Minimum inhibitory concentrations (MIC) were determined as the lowest concentrations showing no visible bacterial growth^53^. In addition, the antibacterial effect of both extracts (cinnamon and ginger extracts) and AgNO_3_ was tested against enterococcal urinary pathogens.

After determining the MIC, samples from wells that showed no bacterial growth were cultivated on brain heart agar and incubated at 37 °C for 24 h. The lowest concentration that reduced the bacterial count by 99.99% compared to the control was considered as minimum bactericidal concentration (MBC).

### Antibiofilm assay

#### Microtiter plate assay

After determining the MIC, values of 1/2, 1/4, and 1/8 MIC (sub- MIC) were used to investigate the antibiofilm activities of the AgNPs. In addition, the antibiofilm activity of the AgNO_3_, ginger and cinnamon extracts was evaluated at sub-MIC values. The assay was carried out as described previously in the crystal violet assay. Wells containing AgNPs free culture medium was used as control.

### Antibiofilm effect of AgNPs on the catheter

With some modification to the LewisOscar et al*.* method, a sterile Foley urinary catheter (Sumbow, China) was cut. Every 3 segments were placed in Wasserman tubes containing 1 ml of TSBG with serial dilution of different AgNPs. Wasserman tubes were then inoculated with 1 ml of adjusted bacterial suspensions (10^6^ CFU/ml) and incubated overnight at 37 °C. After incubation, the collected catheters were washed and placed in a tube containing 1 ml sterile PBS. The tubes were sonicated for 30 s 3 times at 20 kHz, and counting of the released cells was carried out by viable count^54^. Catheters immersed in AgNPs free culture medium was used as control.

### Scanning electron microscope (SEM) of catheters

The antibiofilm effect of the ginger and chemical AgNPs was confirmed by visualization of the biofilm formed on the treated catheters by SEM. Briefly, catheters treated with 1/2 and 1/4 MIC were washed with PBS, and the bacterial cells were fixed with 2% glutaraldehyde and 0.1 M cacodylate for 2 h. The fixed samples were washed three times and dehydrated by passing through serial dilutions of ethanol (50%, 70%, 80%, 90%, and 100%) each for 10 min. The samples were left to dry at room temperature, coated with gold, and examined under the SEM (Thermo Fisher Scientific, USA). Catheter segments in non-inoculated medium and AgNPs free culture medium were used as negative and positive controls, respectively^55^.

### Statistical analysis

Statistical analysis (Student's *t*-test and two-way ANOVA tests) was performed by using GraphPad Prism (GraphPad Soſtware Tools, Inc., La Jolla, CA, USA) to test the effect of cinnamon, ginger, and chemically synthesized AgNPs at different concentrations on the biofilm formation of the enterococcal isolates. Data were presented as mean ± standard deviation, and a *p*-value less than 0.05 was considered statistically significant.

## Supplementary Information

Supplementary Figures.

## Data Availability

All data and results are available upon request. All methods were carried out in accordance with relevant guidelines and regulations. All experimental protocols were approved by the faculty of Pharmacy of The British University in Egypt and Ain Shams University. Informed consent was obtained from all subjects.
