# Supplementary material for: Antibiofilm activity of green synthesized silver nanoparticles against biofilm associated enterococcal urinary pathogens
Source: Sci Rep. 2022 Mar 9;12:3869. doi: 10.1038/s41598-022-07831-y (PMC8907169; doi:10.1038/s41598-022-07831-y)
Supplement: Supplementary file 1 — Supplementary Figures. [file 41598_2022_7831_MOESM1_ESM.pdf]

Antibiofilm Activity of Green Synthesized Silver Nanoparticles Against Biofilm Associated Enterococcal Urinary Pathogens

Nada S. Swidan[1], Yomna A. Hashem[1], Walid F. Elkhatib[2,3], Mahmoud A. Yassien[2*]

[1] Microbiology and Immunology, Faculty of Pharmacy, The British University in Egypt, Cairo, Egypt.

[2] Microbiology and Immunology Department, Faculty of Pharmacy, Ain Shams University, African Union Organization St., Abbassia, Cairo 11566, Egypt.

[3] Department of Microbiology & Immunology, Faculty of Pharmacy, Galala University, New Galala city, Suez, Egypt.

Supplementary Fig. S1

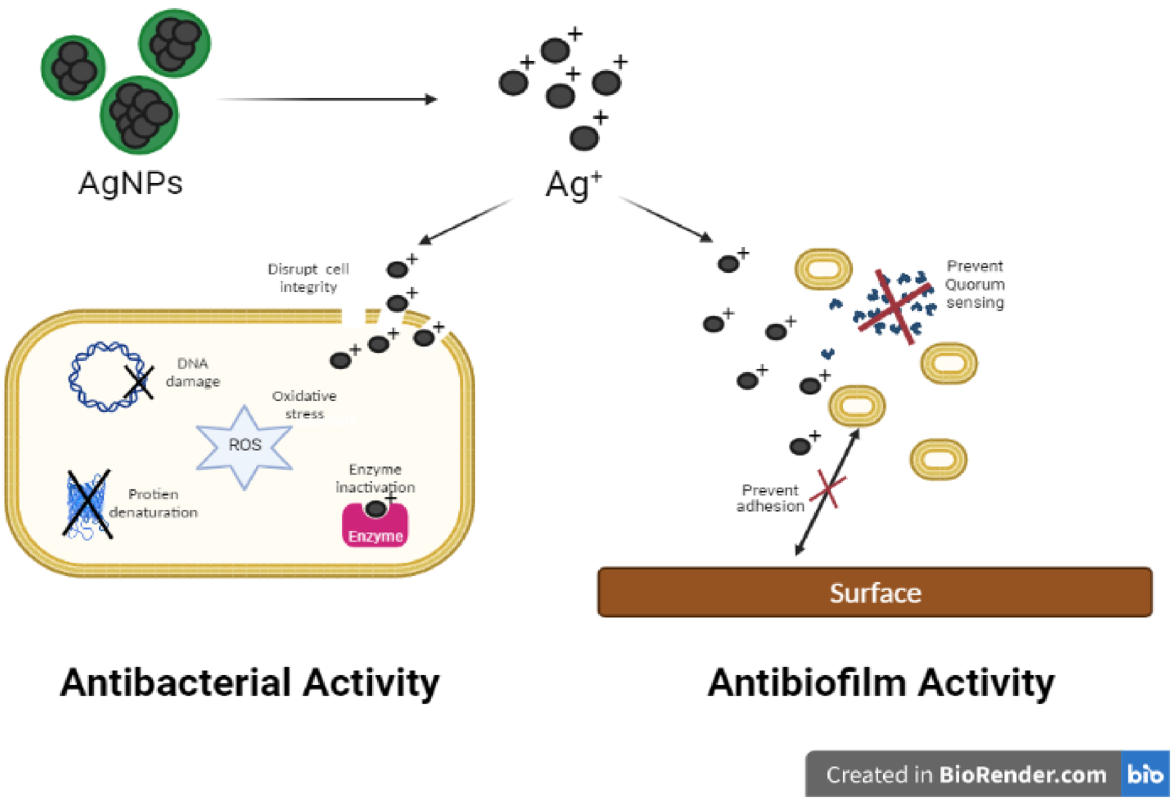

Supplementary Fig. S1 A schematic diagram showing the expected antibacterial and antibiofilm activity of AgNPs.

Supplementary Fig. S2

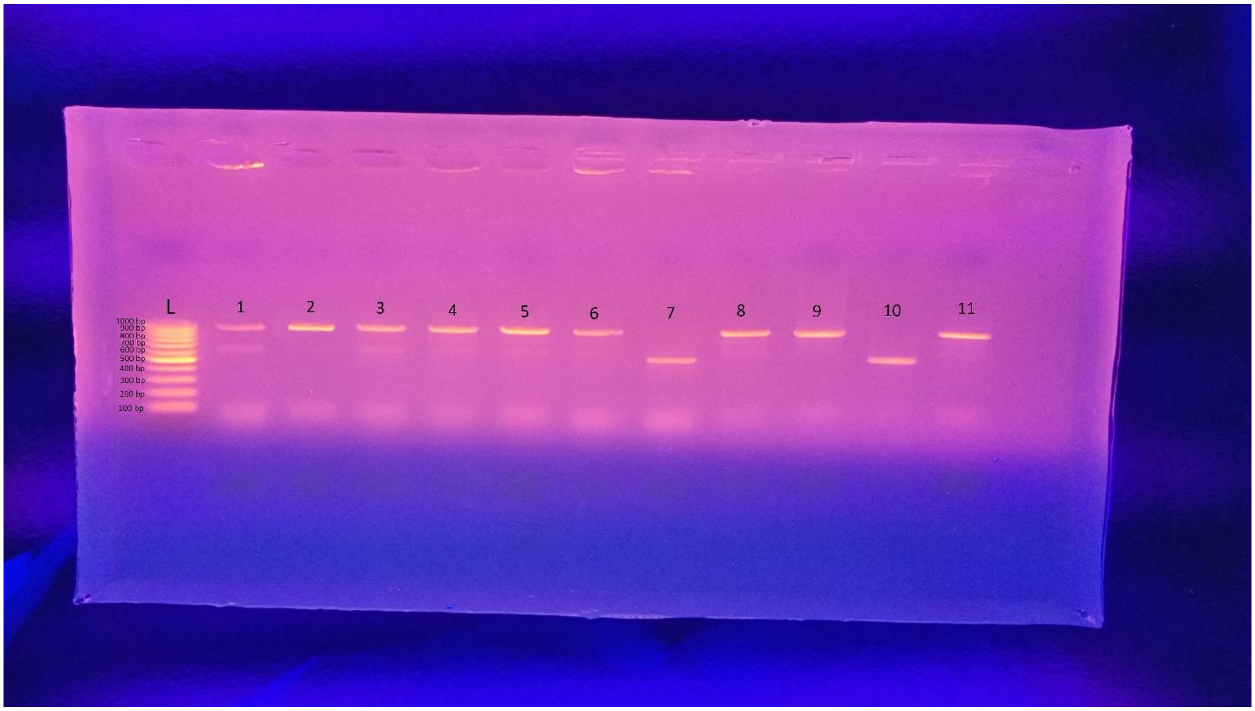

Supplementary Fig. S2 PCR amplification of *ddl E. faecalis* and *ddl E. faecium*. L: 100bp DNA marker, Lane 1,2,3,4,5,6,8,9, and 11: *ddl* gene (942bp) for the detection of *E. faecalis*, Lane 7 and 10: *ddl* gene (535bp) for the detection of *E. faecium*.

Supplementary Fig. S3

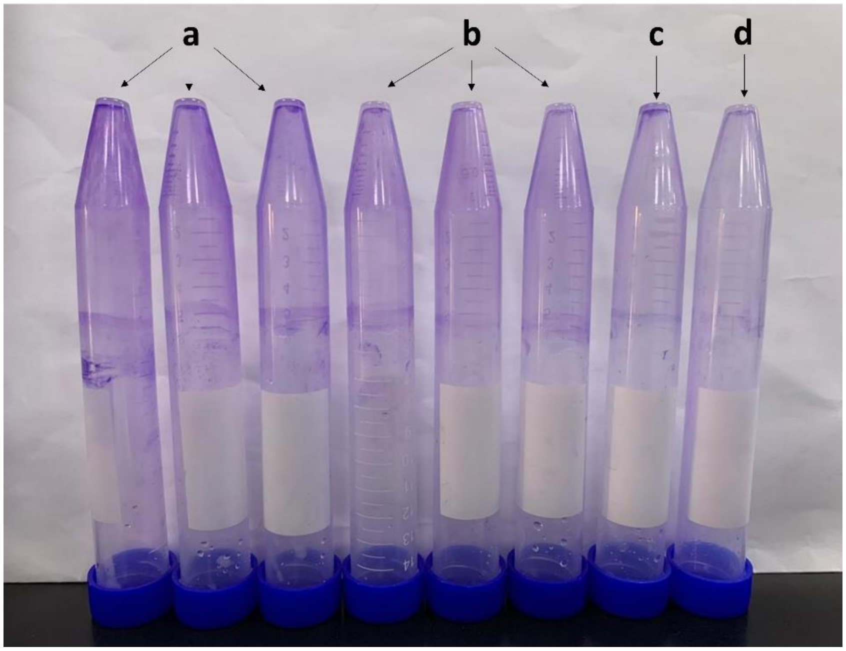

Supplementary Fig. S3 Tube assay image showing the different intensity of biofilm formation by enterococcal isolates where, (a) is strong biofilm, (b) is moderate biofilm, (c) is weak biofilm, and (d) is non-biofilm formation.

Supplementary Fig. S4

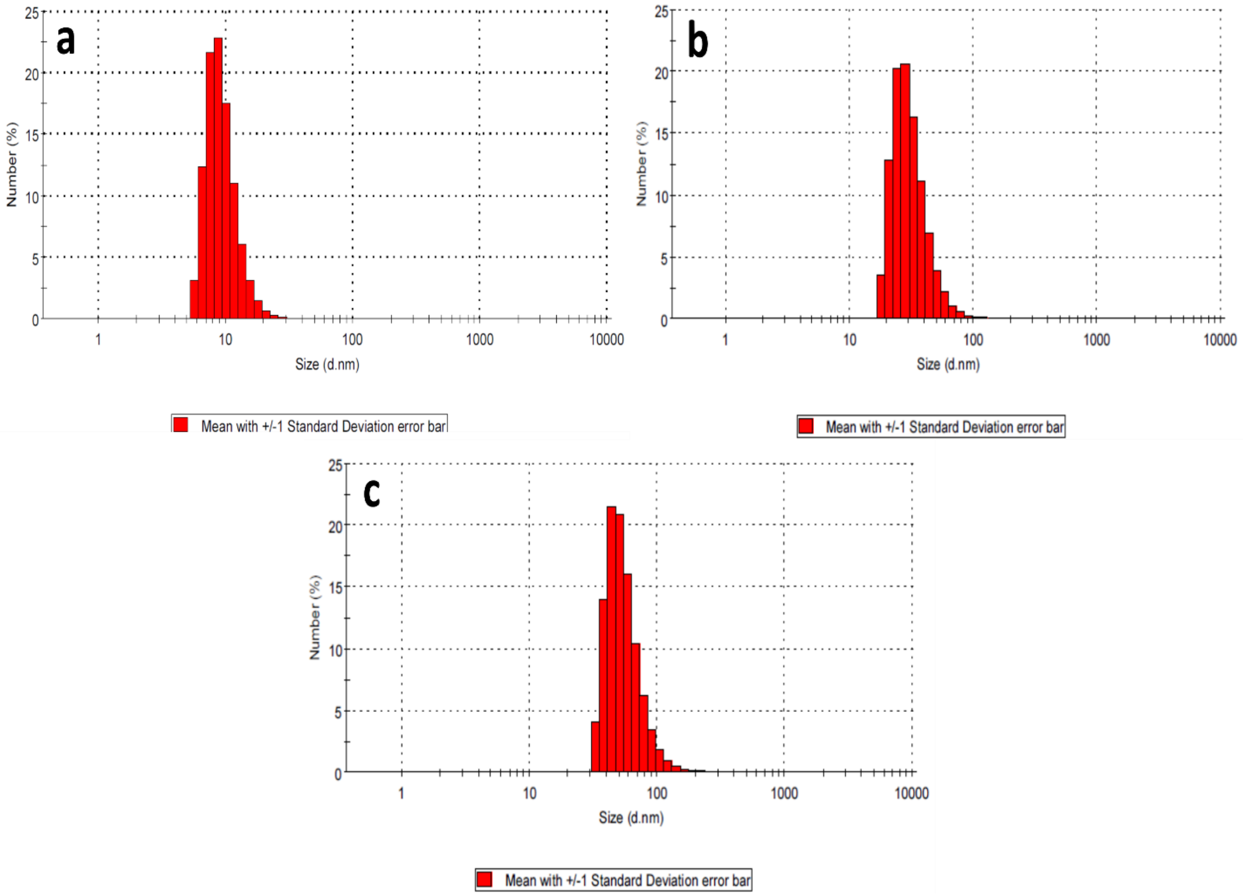

Supplementary Fig. S4 Histogram analysis of particle sizes of (a) cinnamon AgNPs, (b) ginger AgNPs, and (c) chemical AgNPs.

Supplementary Fig. S5

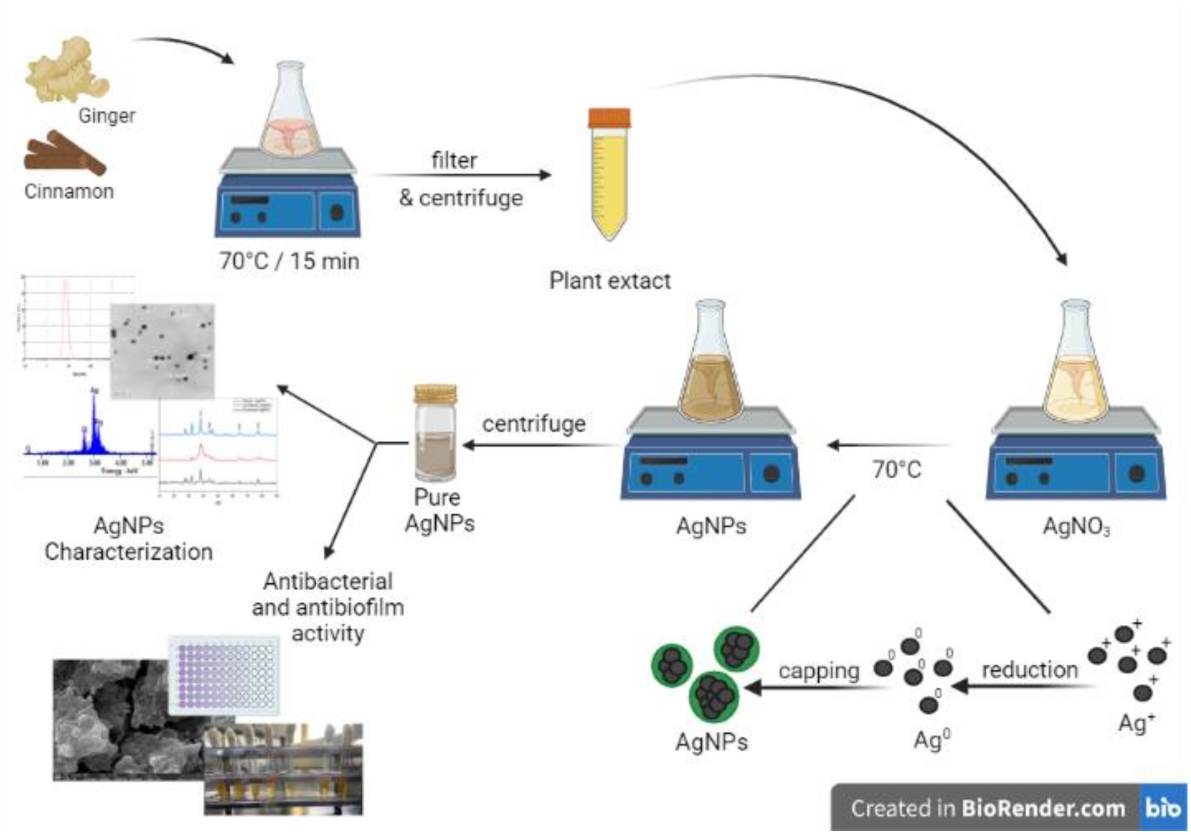

Supplementary Fig. S5 A schematic diagram showing steps and mechanism of cinnamon and ginger AgNPs.
